# Interleukin-1beta and tumour necrosis factor-alpha impede neutral lipid turnover in macrophage-derived foam cells

**DOI:** 10.1186/1471-2172-9-70

**Published:** 2008-11-25

**Authors:** Jenny Persson, Jan Nilsson, Marie W Lindholm

**Affiliations:** 1 Department of Clinical Sciences Malmö, Clinical Research Center, UMAS, Lund University, Sweden

## Abstract

**Background:**

Pro-inflammatory cytokines can affect intracellular lipid metabolism. A variety of effects have been described for different cell types; hepatocyte lipid turnover pathways are inhibited during inflammation, whereas interleukin-1β (IL-1β) reduces intracellular cholesterol levels in fibroblasts. Levels of the pro-inflammatory cytokines IL-1β and tumour necrosis factor-α (TNF-α) are up-regulated at sites of formation of atherosclerotic plaques. Plaque formation is though to begin with infiltration of monocytes to the intimal layer of the vascular wall, followed by differentiation to macrophages and macrophage uptake of modified lipoproteins, resulting in accumulation of intracellular lipids. The lipid-filled cells are referred to as macrophage foam cells, a key feature of atherosclerotic plaques. We have investigated the effects of IL-1β and TNF-α on macrophage foam cells in order to assess whether presence of the pro-inflammatory cytokines improves or aggravates macrophage foam cell formation by affecting lipid accumulation and lipid turn-over in the cells.

**Results:**

Differentiated primary human macrophages or THP-1 cells were lipid loaded by uptake of aggregated low density lipoproteins (AgLDL) or very low density lipoproteins (VLDL), and then incubated with IL-1β (0 – 5000 pg/ml) in lipoprotein-free media for 24 h. Cells incubated in absence of cytokine utilized accumulated neutral lipids, in particular triglycerides. Addition of exogenous IL-1β resulted in a dose-dependent retention of intracellular cholesterol and triglycerides. Exchanging IL-1β with TNF-α gave a similar response. Analysis of fatty acid efflux and intracellular fatty acid activation revealed a pattern of decreased lipid utilization in cytokine-stimulated cells.

**Conclusion:**

IL-1β and TNF-α enhance macrophage foam cell formation, in part by inhibition of macrophage intracellular lipid catabolism. If present *in vivo*, these mechanisms will further augment the pro-atherogenic properties of the two cytokines.

## Background

Human lipid and lipoprotein metabolism is altered during acute phase response. A striking example is sepsis (endotoxinemia), which is correlated with severe hypertriglyceridemia. In cells such as hepatocytes, smooth muscle cells, and adipocytes several effects of endotoxins on intracellular lipid metabolism are identical to, or mediated by, effects of pro-inflammatory cytokines such as interleukin-1β (IL-1β) and tumour necrosis factor-α (TNF-α) [1]. These two cytokines are also present in atherosclerotic plaques, where it has been suggested that the cytokines mediate a constant low-grade inflammatory state. If they affect lipid metabolism in cells in the vascular wall in a similar manner as they affect peripheral tissues during acute phase response, such effects may contribute to atherosclerotic plaque development.

Macrophage foam cells are a characteristic feature of atherosclerotic plaques. During foam cell formation modified lipoproteins enters cells by receptor mediated uptake and excess neutral lipids are stored as lipid droplets, creating a typical foamy appearance. Oxidized forms of low density lipoproteins (LDL) are the modified lipoprotein usually considered causative during foam cell formation *in vivo*, but excessive uptake of other lipoproteins can also result in intracellular lipid accumulation and give macrophages a foamy appearance. We have previously described an *in vitro *model system for foam cell formation, where cells are lipid loaded by incubation with aggregated LDL (AgLDL) or very low density lipoproteins (VLDL). In this *in vitro *model system all cells incubated with AgLDL or VLDL have large numbers of intracellular lipid droplets, illustrating a model for macrophage foam cell formation with predominantly cholesterol ester or triglyceride rich lipid droplets, respectively [2].

We hypothesised that IL-1β and TNF-α affect lipid turnover in macrophage foam cells, a mechanism not previously described for this cell type. After lipid loading, foam cells were treated with either cytokine, mimicking the *in vivo *situation where atherosclerosis development is propagated by both hyperlipidemia and inflammation.

## Results

### Intracellular lipid content of macrophage foam cells

Isolated primary human monocytes were differentiated to macrophages and lipid loaded by incubation with VLDL or AgLDL. Control cells were incubated for the same period of time in lipoprotein-free cell culture media. After incubation in presence or absence of lipoproteins all cells were rinsed with heparin in order to remove lipoproteins attached to the cell surface. Fresh cell culture media supplemented with 0–5000 pg cytokine/ml was added to cells and the macrophages were incubated in this media for an additional 24 h. Cell viability was not affected by this treatment, as judged by two different assays performed both directly after lipoprotein lipid loading and after subsequent cytokine treatment.

**Figure 1:**
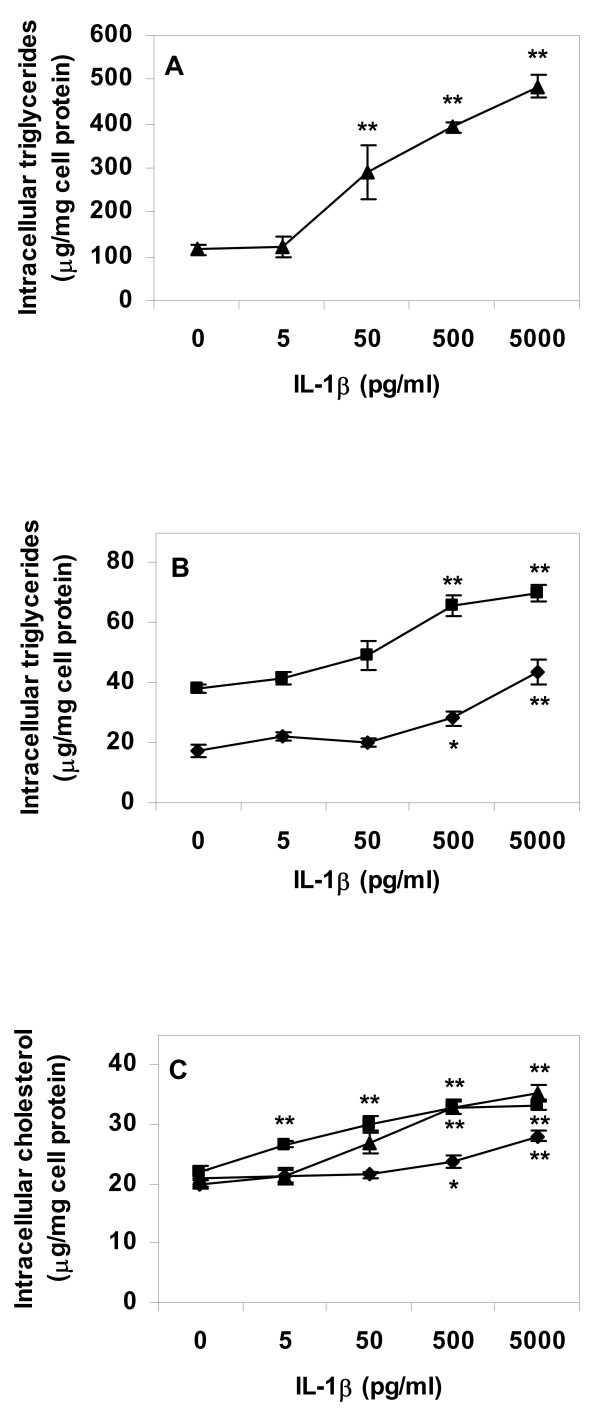
**Lipid content of primary human macrophage incubated with IL-1β**. Cells were differentiated for four days with GM-CSF, and then incubated for 24 h in absence or presence of lipoproteins (50 μg/ml), rinsed with heparin and incubated with IL-1β in lipoprotein-free media for an additional 24 h. Intracellular lipids were extracted and lipid values normalized to cell protein content. A. Triglyceride content of VLDL treated cells, B. Triglyceride content of control or AgLDL treated cells, C. Cholesterol content of cells. Data represents mean ± SEM (n = 6) for a representative experiment repeated three times with cells from different donors. * = P < 0.05, ** = P < 0.01 compared to cells incubated in absence of IL-1β. Diamonds = control cells (no lipoprotein lipid loading), squares = AgLDL, triangles = VLDL.

**Figure 2:**
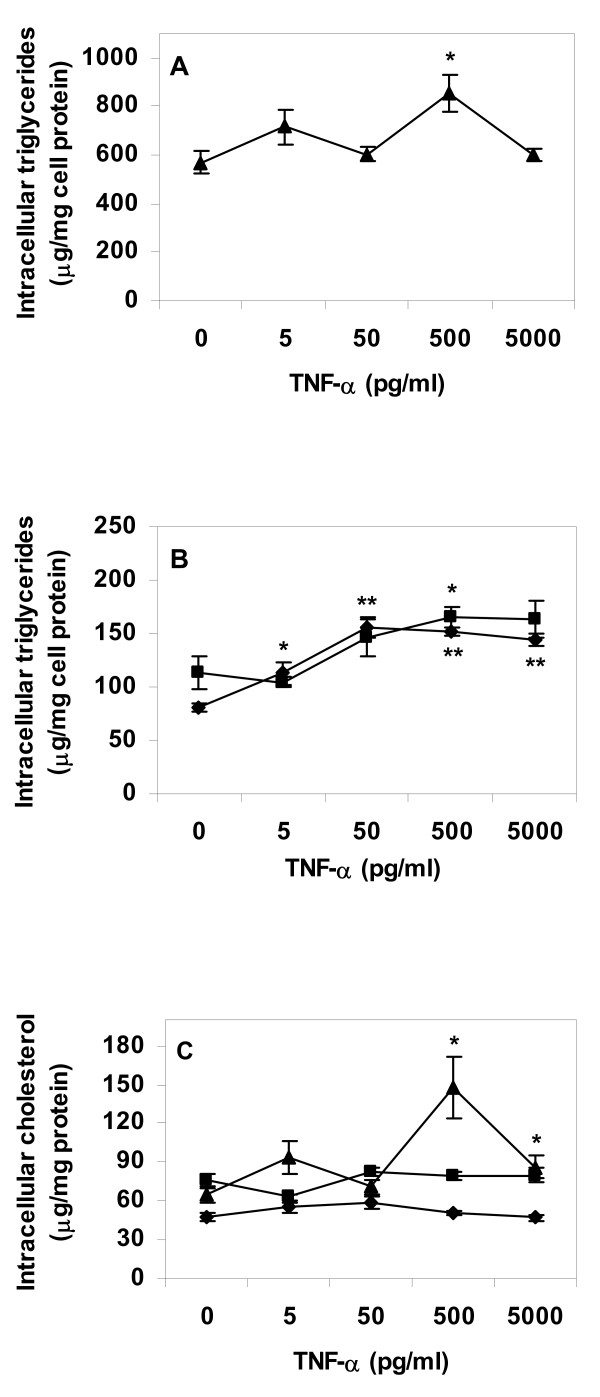
**Lipid content of primary human macrophage incubated with TNF-α**. Cells were differentiated for four days with GM-CSF, and then incubated for 24 h in absence or presence of lipoproteins (50 μg/ml), rinsed with heparin and incubated with TNF-α in lipoprotein-free media for an additional 24 h. Intracellular lipids were extracted and lipid values normalized to cell protein content. A. Triglyceride content of VLDL treated cells, B. Triglyceride content of control or AgLDL treated cells, C. Cholesterol content of cells. Data represents mean ± SEM (n = 6) for a representative experiment repeated three times with cells from different donors. * = P < 0.05, ** = P < 0.01 compared to cells incubated in absence of IL-1β. Diamonds = control cells (no lipoprotein lipid loading), squares = AgLDL, triangles = VLDL.

Incubation of control macrophages and macrophage foam cells with IL-1β resulted in higher levels of intracellular lipids in cytokine treated cells, in a cytokine dose-dependent manner. Effects were most pronounced in cells lipid loaded by incubation with VLDL before cytokine treatment (Fig. 1A and 1C). The same pattern was apparent in control cells and cells incubated with AgLDL before cytokine treatment (Fig. 1B and 1C). Experiments were repeated with TNF-α replacing IL-1β. TNF-α induced a dose-dependent increase in intracellular triglyceride levels of control and AgLDL treated cells, whereas there was a limited effect of TNF-α on VLDL treated cells (Fig. 2A and 2B). TNF-α had no strong effect on intracellular cholesterol levels neither in lipid loaded nor in control cells (Fig. 2C).

**Figure 3:**
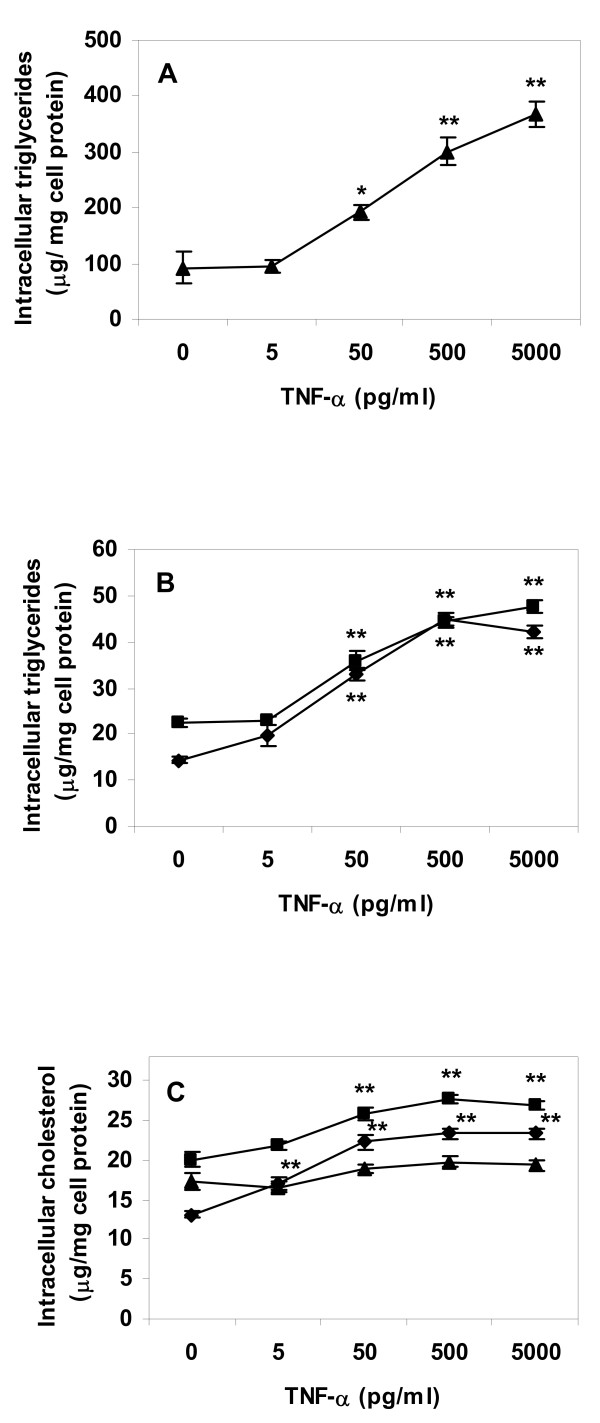
**Lipid content of THP-1 macrophages incubated with TNF-α**. Cells were differentiated for four days with PMA, and then incubated for 24 h in absence or presence of lipoproteins (50 μg/ml), rinsed with heparin and incubated with TNF-α in lipoprotein-free media for an additional 24 h. Intracellular lipids were extracted and lipid values normalized to cell protein content. A. Triglyceride content of VLDL treated cells, B. Triglyceride content of control or AgLDL treated cells, C. Cholesterol content of cells. Data represents mean ± SEM (n = 6) for a representative experiment repeated three times with cells from different donors. * = P < 0.05, ** = P < 0.01 compared to cells incubated in absence of TNF-α. Diamonds = control cells (no lipoprotein lipid loading), squares = AgLDL, triangles = VLDL.

**Figure 4:**
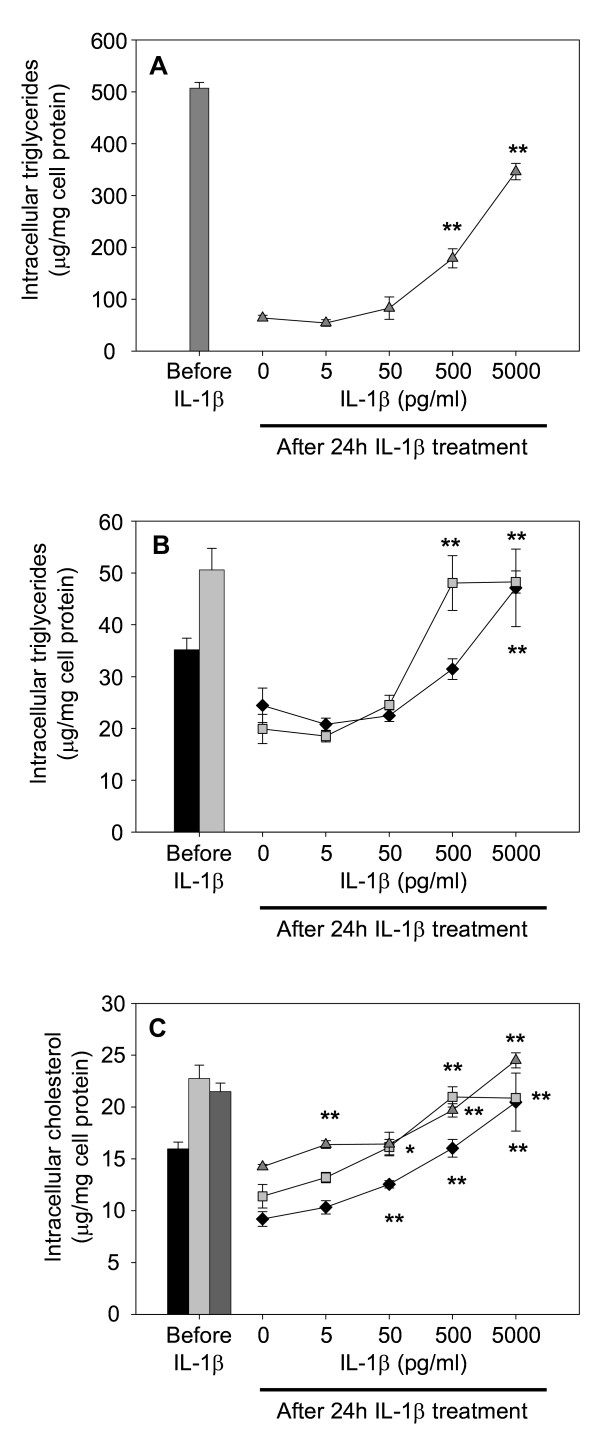
**Lipid content of THP-1 macrophages incubated with IL-1β**. Cells were differentiated for four days with PMA, and then incubated for 24 h in absence or presence of lipoproteins (50 μg/ml), rinsed with heparin and incubated with IL-1β in lipoprotein-free media for an additional 24 h. Intracellular lipids were extracted and lipid values normalized to cell protein content. A. Triglyceride content of VLDL treated cells, B. Triglyceride content of control or AgLDL treated cells, C. Cholesterol content of cells. Data represents mean ± SEM (n = 6) for a representative experiment repeated three times with cells from different donors. * = P < 0.05, ** = P < 0.01 compared to cells incubated in absence of IL-1β. Diamonds = control cells (no lipoprotein lipid loading), squares = AgLDL, triangles = VLDL.

The experiments were repeated using the human leukaemia cell line THP-1. In this cell type TNF-α displayed a strong and significant effect on intracellular lipid levels in lipid loaded cells as well as in control cells. Exposure to the cytokine resulted in high levels of intracellular triglyceride and total cholesterol regardless of lipid loading before cytokine treatment (Fig. 3). The same was apparent after incubation of THP-1 cells with IL-1β (Fig. 4). Included in Fig. 4 are intracellular lipid levels directly after lipoprotein lipid loading, i.e. before exposure to cytokine. Incubation of lipid loaded cells with 5000 pg IL-1β/ml, i.e. the highest concentration used in our experiments, resulted in intracellular lipid values close to levels before cytokine treatment (Fig. 4). This suggests that the cytokines suppress macrophage lipid turnover. We therefore proceeded to analyze whether IL-1β and TNF-α affected mechanisms for lipid efflux and lipid metabolism in macrophage foam cells.

### Fatty acid efflux and triglyceride turn-over

**Figure 5:**
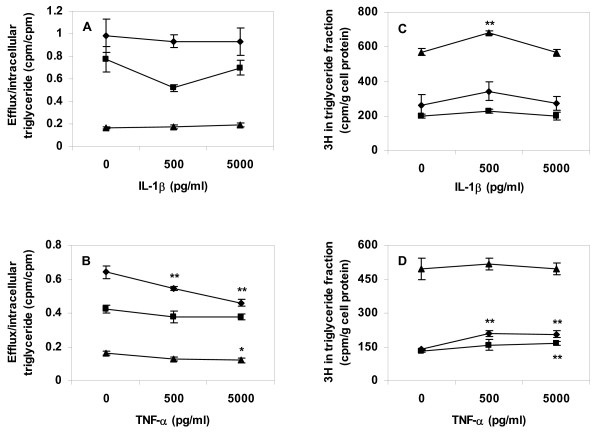
**Fatty acid efflux and triglyceride lipid label in cytokine treated THP-1 cells**. Cells were differentiated for four days, then incubated for 24 h with [9,10(n)-^3^H]-oleate pre-incubated with lipoproteins (50 μg/ml) or added directly to cells (1 μCi/well). After removing non-attached lipids with a heparin rinse, cells were incubated in lipoprotein-free media for an additional 24 h with 0, 500, or 5000 pg/ml of respective cytokine. Cell lipids were extracted and lipid extracts separated by thin layer chromatography. Label was quantified in intracellular triglyceride fractions and conditioned media. A. Fatty acid label efflux normalized to intracellular triglyceride label in IL-1β treated cells, B. Fatty acid label efflux normalized to intracellular triglyceride label in TNF-α treated cells, C. Fatty acid label in intracellular triglyceride fractions from IL-1β treated cells, D. Fatty acid label in intracellular triglyceride fractions from TNF-α treated cells. Data represents mean ± SD (n = 6) for a representative experiment repeated three times. * = P < 0.05, ** = P < 0.01, *** = P < 0.001. Diamonds = control cells (no lipoprotein lipid loading), squares = AgLDL, triangles = VLDL.

THP-1 cells were incubated in presence or absence of lipoproteins and thereafter incubated with 0–5000 pg cytokine/ml. Conditioned media after cytokine treatment were analyzed for free fatty acid (FFA) content by colorimetric analysis. After cytokine treatment, FFA levels were 3–4 times higher in conditioned media from triglyceride-rich (VLDL treated) cells than in media from cholesterol-rich (AgLDL treated) cells or in conditioned media from cells incubated in absence of lipoproteins, however there was no significant correlation between cytokine concentration and FFA levels in conditioned media from any cell type (data not shown). In an additional series of experiment THP-1 cells were labelled with [9,10(n)-^3^H]-oleate during lipoprotein uptake. Cells were then treated with either of the two cytokines in lipoprotein and label free media. Exposure of labelled and lipid loaded cells to TNF-α significantly decreased the amount of fatty acid label recovered in conditioned media (Fig. 5A and 5B). Cytokine treatment of cells also resulted in increased retention of labelled fatty acid in the intracellular triglyceride (lipid droplet) fraction (Fig. 5C and 5D).

### Acyl-Coenzyme A synthetase activity

**Figure 6:**
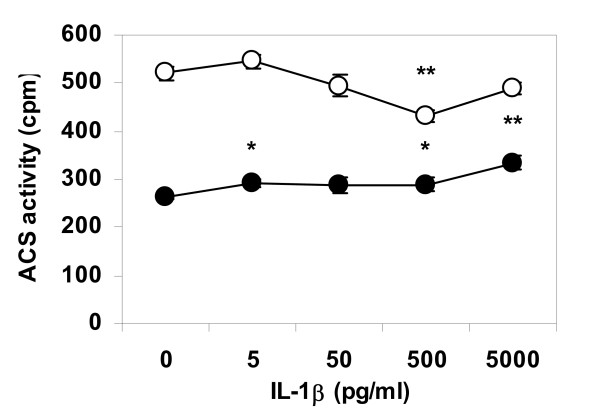
**Acyl-CoA activity in triglyceride-loaded THP-1 cells treated with IL-1β**. Cells were differentiated, incubated for 24 h with VLDL (50 μg/ml), and then incubated in lipoprotein-free media for an additional 24 h with IL-1β. Cells were homogenized and intracellular fractions isolated by differential centrifugation. Enzymatic activity during a five minutes incubation was assayed in fractions containing mitochondria (open circles) or endoplasmic reticulum (closed circles). Data represents mean ± SEM, n = 6, for a representative experiment repeated three times. * = P < 0.05, ** = P < 0.01.

Differentiated THP-1 cells were lipid loaded by incubation with VLDL, and then treated with IL-1β in lipoprotein free media as described above. Cells were homogenized and separated in sub-cellular fractions. Acyl-CoA synthetase activity was assessed for five minutes in aliquots containing mitochondria or endoplasmic reticulum. Enzymatic activity was affected by cytokine treatment in a dose-dependent manner, where IL-1β stimulated acyl-CoA synthetase activity of the endoplasmic reticulum and suppressed enzyme activity in mitochondria, indicating enhanced fatty acid esterification and decreased β-oxidation in cytokine-stimulated cells (Fig 6).

### Expression and secretion of apolipoprotein E

**Figure 7:**
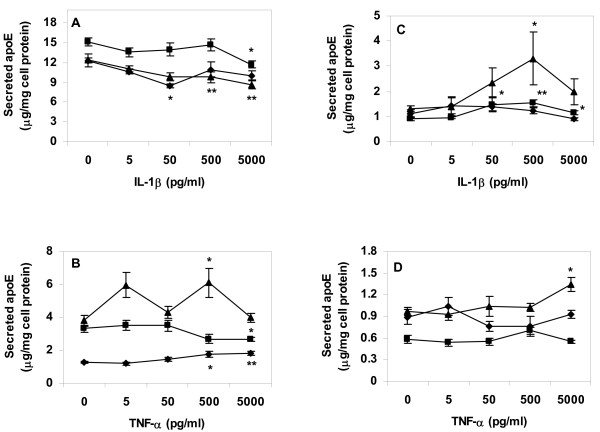
**Apolipoprotein E secretion from lipid loaded and cytokine treated cells**. Cells were differentiated for four days, lipid loaded for 24 h, then treated with respective cytokine in lipoprotein-free media for an additional 24 h. ApoE content was measured in conditioned media after cytokine treatment. A. ApoE secreted from primary human macrophages after lipid loading and treatment with IL-1β. B. ApoE secreted from primary human macrophages after lipid loading and treatment with TNF-α. C. ApoE secreted from THP-1 cells after lipid loading and treatment with IL-1β. D. ApoE secreted from THP-1 cells after lipid loading and treatment with TNF-α. Data represents mean ± SEM (n = 6) for a representative experiment repeated three times. * = P < 0.05, ** = P < 0.01 compared to cells incubated in absence of TNF-α. Diamonds = control cells (no lipoprotein lipid loading), squares = AgLDL, triangles = VLDL.

Primary human macrophages or THP-1 cells were incubated in absence of lipoproteins, or with VLDL or AgLDL, and then treated with IL-1β or TNF-α as in previous experiments. Incubation with either cytokine did not result in any consistent effects on apolipoprotein E (apoE) secretion from cells (Fig. 7). Real-time PCR analysis did not demonstrated any significant effects of either cytokine on apoE mRNA levels in lipid loaded or control cells (data not shown).

## Discussion

Atherosclerosis is a disease accelerated by inflammation and by plasma dyslipidemia, where the latter typically include high levels of LDL and VLDL. In virtually all eukaryotic cells intracellular lipid will accumulate in lipid droplets when lipoprotein lipid uptake exceeds lipid degradation and efflux. In atherosclerotic plaques, macrophage foam cell lipid droplets mainly accumulate cholesterol ester [3]. Pro-inflammatory cytokines have earlier been reported to increase intracellular cholesterol levels in some cell types; IL-1β increases cholesterol accumulation in mesengial cells by up-regulation of scavenger receptors, by dysregulation of the LDL receptor, and by inhibition of cholesterol efflux mediated by the ATP binding cassette transporter protein A1 (ABC-A1) [4]. Cytokines have also been reported to affect macrophage lipid efflux in mouse J774 cells, where IL-1β or TNF-α both decrease mRNA and protein levels of ABC-A1 [5].

It is clear from our experiments that when THP-1 cells were lipid loaded by incubation with lipoproteins and then incubated in cytokine free media, the cells utilized stored lipids (Fig. 4: the "0" points for individual dose-response curves compared to respective intracellular lipid value before incubation with IL-1β). Cytokine treatment after lipoprotein lipid loading resulted in dose-dependent retention of intracellular lipid. The results suggest that increased cellular lipid content after incubation with the pro-inflammatory cytokine is the result of decreased intracellular lipid catabolism and/or efflux. However, we cannot exclude that lipid content of cells after cytokine treatment in part also may reflect affected lipid synthesis. Such directs effects on lipid synthesis have been reported for adipocytes, where TNF-α decreases *de novo *fatty acid synthesis, whereas IL-1β increases it [6].

Macrophage foam cells lack pathways for cholesterol degradation; excess lipid can only be removed from cells via reverse cholesterol transport pathways. Excess cholesterol is removed from macrophage foam cells as oxysterols, via efflux to extra-cellular acceptor particles, or via endogenous production of apoE leading to formation of LpE particles that mediate cholesterol removal from human monocyte-derived macrophages in absence of serum or exogenous acceptor particles [7,8]. Cytokine treatment may directly affect apoE production; long-term (3 days) treatment with TNF-α stimulates human monocyte (but not macrophage) apoE mRNA expression and protein levels *in vitro *[9]. This effect may be species-specific, as 2 days of incubation with TNF-α or IL-1β decreased *de novo *synthesis of apoE secretion in mouse peritoneal macrophages to approximately half of the level in control cells [10]. Neither cytokine exerted any consistent effect on apoE secretion from control or lipid-filled cells. Our experiments were performed in absence of extra-cellular acceptor particles, as we aimed to analyze cytokine effects on endogenous macrophage lipid efflux pathways only. Additional experiments using the same model systems as above in presence of exogenous apoA-I or HDL particles will shed further light on whether cytokine treatment also affect lipid efflux pathways involving ABC transporters.

Treatment of macrophage foam cells with IL-1β or TNF-α may decrease overall foam cell lipid efflux capacity, although our data on efflux of labelled fatty acids did not demonstrate cytokine effects strong enough to account for the entire effect on intracellular lipid retention. TNF-α (but not IL-1β) decreased efflux of labelled fatty acid from cells, and both cytokines showed trends towards increasing the amount of fatty acid label retained in the intracellular triglyceride fraction after lipid loading and cytokine treatment. These data indicate that cytokine treatment may impede foam cell lipid droplet turnover.

An interesting observation is that regardless of method of lipid loading cytokine effects on neutral lipid turnover appeared to be stronger or more pronounced for triglycerides than for cholesterol ester. Foam cell lipid droplet turnover of triglycerides has been reported to be three to four times faster than that of cholesterol esters [11]. It is likely that the effects of IL-1β and TNF-α are partially mediated by cytokine effects on enzymatic activity of acyl-CoA synthetase, a key point for fatty acid oxidation but also for fatty acid esterification and storage in triglycerides, cholesterol ester, and phospholipids. Enzymatic activity depends not only on protein levels or substrate availability but also on intracellular location, as the same enzyme activates fatty acids for catabolic β-oxidation in mitochondria as for anabolic esterification on the endoplasmic reticulum. For that reason, we chose to analyze acyl-CoA synthetase activity in different subcellular fractions rather than in whole cell homogenates.

It has to be pointed out that Figure 6 illustrates ACS enzyme activity during five minutes of incubation only. The short incubation time of the ACS assay is necessary for a linear dose response ratio between enzyme activity and production of labelled product. The effects of IL-1β and TNF-α on total intracellular triglyceride and cholesterol content illustrated in Figures 1, 2, 3, 4 are the result of incubations with respective cytokine for 24 h. Therefore, although the cytokine effect on ACS activity during short term incubation is minor, it is likely that the combined decrease in activation of fatty acids for subsequent β-oxidation and increase in activation of fatty acids for esterification will result in major accumulation of intracellular lipids over time.

It can be argued that cytokine concentrations used in our experiments are high compared to what is found *in vivo*. Serum concentrations of IL-1β and TNF-α are typically in the low pg/ml range, with increases in serum concentrations found in post-infarction and diabetic patient (values ranging between 10–25 pg/ml). It is well documented that IL-1β and TNF-α expression is strongly increased in atherosclerotic plaques compared with surrounding tissues, but this is typically analyzed with immunohistochemical methods or with PCR analysis of mRNA expression [12,13]. Attempts have been made to analyze plaque cytokine production directly, such as analyses of TNF-α secretion in endotoxin-stimulated culture of explanted human atherosclerotic plaque tissue [14], with final cytokine concentration in conditioned media reaching 200 pg/ml. It is likely that concentrations of cytokines in the vicinity of cells in atherosclerotic plaques are several degrees of magnitude higher than serum concentrations or concentrations in tissue culture, making the higher concentrations used in the current experiment possible *in vivo*.

## Conclusion

Our data suggests that IL-1β and TNF-α may share a previously unknown effect on macrophage foam cells, resulting in retention of neutral lipids by a combination of decreased lipid efflux, decreased β-oxidation substrate availability, and stimulated fatty acid esterification. If such mechanisms are active *in vivo*, the high neutral lipid content of cytokine-treated cells may contribute to atherosclerotic development and even accelerate development of unstable lipid-rich atherosclerotic plaques.

## Methods

### Lipoprotein isolation

Lipoproteins were prepared by differential ultra centrifugation of fresh plasma from healthy donors [15]. Lipoproteins were mixed with sucrose (10%, w/v) and EDTA (4.8 mM, pH = 7.4), frozen immediately and stored at -80°C [16]. Just before use, lipoproteins were desalted and lipoprotein concentration determined with BCA (bicinchoninic acid) protein assay reagent (Pierce). AgLDL was prepared by vortex for 3 min [17].

### Isolation and culture of primary human monocytes/macrophages

Human peripheral blood monocytes were isolated from buffy coats obtained from healthy blood donors as previously described [18]. Cells were differentiated for four days in macrophage-serum free media with granulocyte macrophage-colony stimulating factor (GM-CSF, 1 ng/ml) [19]. After this, fresh GM-CSF free media was added to each plate. Cells were incubated for 24 h with or without addition of AgLDL or VLDL, both at 50 μg protein/ml. After lipid loading cells were washed with a heparin solution (20 U/ml in PBS) in order to remove lipoproteins attached to cell surfaces. Finally, cells were incubated for an additional 24 h in absence of lipoproteins with addition of IL-1β or TNF-α (0–5000 ng/ml, cell culture tested, BD Biosciences), as specified in the Result section.

### THP-1 cell culture

Human THP-1 monocytes were differentiated with PMA for four days in accordance with instructions from ECACC (European collection of cell cultures). After this, fresh serum and PMA free media supplemented with BSA (1%) was added to cells. As for primary cells, THP-1 cells were incubated for 24 h with or without addition of AgLDL or VLDL, both at 50 μg protein/ml. After lipid loading, cells were subjected to the same heparin rinse as primary cells, and then incubated for an additional 24 h in serum, lipoprotein, and PMA free media with addition of 1% BSA and IL-1β or TNF-α (0–5000 ng/ml), as specified in the Result section.

### Cell viability

Lipoprotein and cytokine treatments at concentrations used in the experiments were not toxic to either cell type, as judged by the methylthiazoletetrazolium assay (Sigma) or leakage of lactate dehydrogenase (Sigma assay) to cell culture media.

### Cell lipid and protein analyses

Cell lipids were extracted with hexane:isopropanol (3:2 + 0.005% butylated hydroxytoluene). Lipid analyses (triglycerides and total cholesterol) were performed with colorimetric assays (Wako). After lipid extraction, residual cell proteins were dissolved in 0.3 M NaOH (*aq*) and cell lysates used for cell protein analyses with BCA protein assay reagent.

### Analysis of free fatty acid content

FFA content of conditioned cell culture media was analyzed by a colorimetric method according to the manufacturer's instructions (Wako NEFA C ACS-ACOD) using a 0–1 mM serial dilution of stabilized oleate as standard, prepared fresh for each analysis.

### Fatty acid turnover

Differentiated THP-1 cells were incubated for 24 h in serum and PMA free media in absence or presence of lipoprotein (50 μg/ml). Labelled fatty acid ([9,10(n)-^3^H]-oleate in ethanol, 1 μCi/sample) was added either to lipoproteins before addition to cells, or directly to plates of control cells incubated in absence of lipoproteins. Control experiments demonstrated that the lipid label mainly resided in triglyceride, cholesterol ester, and phosphatidyl choline fractions of cells after initial lipoprotein uptake and fatty acid labelling of cells, with only low levels of label recovered in the intracellular FFA fraction. After labelling, cells were rinsed with a heparin solution (20 IU/ml in PBS) and incubated for an additional 24 h in fresh serum, lipoprotein, and PMA free media in presence of 0–5000 pg IL-1β or TNF-α/ml. Efflux of labelled fatty acids and fatty acid degradation products to conditioned media was analyzed by liquid scintillation counting after centrifugation for 5 min at 1000 rpm (to remove floating cells and cell debris). At termination of experiments, cells were rinsed with the heparin solution and cell lipids extracted as described above. Lipid extracts were separated in polar and neutral lipid classes by high performance thin layer chromatography [20]. Triglyceride fractions were extracted from silica with ethanol and analyzed by liquid scintillation counting.

### Acyl-Coenzyme A synthetase activity

Acyl-CoA synthetase activity was assayed according to Tanaka *et al *[21]. Assay conditions were optimized for a linear response between enzyme activity and accumulation of labelled product. Briefly, treated and rinsed THP-1 cells were carefully homogenized in 0.25 M sucrose with handheld miniature homogenizers. Nuclear fractions were removed by repeated homogenization and centrifugation for 10 min at 600*g*. The mitochondrial fraction was separated by centrifugation for 20 min at 13000*g*. Mitochondria and the remaining fraction (containing the endoplasmic reticulum) were incubated for five minutes with [1-^14^C]-palmitate (0.2 μCi/sample) in presence of CoA and ATP. After termination of experiment the remaining labelled fatty acid was separated from labelled acyl-CoA by repeated extractions with n-heptane. Enzymatic activity of acyl-CoA synthetase was finally assessed by scintillation counting of the aqueous fraction, which after n-heptane extraction contains labelled palmitate-CoA only.

### Cytokine ELISA

After incubation of cells, protease inhibitor (phenyl methyl sulfonyl fluoride saturated in isopropanol) was added to conditioned media and samples were spun for 10 min at 2500 rpm in order to remove debris. Samples were either analysed directly or divided in aliquots and stored at -20°C until analysis. Concentrations of secreted endogenous IL-1β and transforming growth factor-β (TGF-β) were analyzed by commercial ELISA sets (R&D systems).

### Apolipoprotein E ELISA

Hi-bond ELISA plates were incubated over night at 4°C with monoclonal IgG_1 _mouse anti-human apoE (Santa Cruz Biotechnology) diluted 1:200 in PBS. Plates were blocked at room temperature for 2 h with 4% BSA in 0.15 M PBS, and then incubated for 2 h at room temperature with samples in duplicates. Human apoE (purified from human plasma VLDL, 0–500 ng/ml, Calbiochem), in non-conditioned serum-free cell culture medum was used as standard. After this, plates were incubated with polyclonal goat anti-human apoE (Calbiochem) diluted 1:8000 in 0.5 M PBS with 0.2% Tween-20 (PBS+T) and 4% BSA for 2 h in room temperature, and then over night at 4°C. Finally, plates were incubated for 2 h at room temperature with biotinylated horse anti-goat IgG (Immunkemi) diluted 1:5000 in PBS+T with 4% BSA, and then for an additional 2 h at room temperature with horse-radish peroxidase conjugated avidin diluted 1:5000 in PBS+T with 4% BSA. ApoE concentrations were quantified with tetramethylbenzidine substrate reagent at 450 nm with 570 nm as reference wavelength.

### ApoE PCR

Total RNA was isolated using RNeasy micro kit following the manufacturer's protocol (Qiagen). RNA was treated with DNAse (deoxyribonuclease I, Sigma) and cDNA synthesis performed according to standard protocol [22]. Primer sequences for human apoE were: forward primer ACTGAGGGCGCTGATGGA, reverse primer GGTCAGTTGTTCCTCCAGTTCC. Real-time PCR was performed using SYBR Green reagent on a Perkin-Elmer ABI Prism 7700 with VLDL-loaded THP-1 cells used for preparation of standard curve and 18S as internal control.

### Statistical analyses

Data are represented as mean ± SEM. Statistical analysis was performed by Mann-Whitney test using SPSS 12.0.1 software. P-values < 0.05 were considered statistically significant.

## List of Abbreviations

ABC-A1: ATP binding cassette transporter protein A1; AgLDL: aggregated low density lipoproteins; apoE: apolipoprotein E; GM-CSF: granulocyte macrophage-colony stimulating factor; IL-1β: interleukin-1β; LDL: low density lipoproteins; TNF-α: tumour necrosis factor-α; TGF-β: transforming growth factor-β; VLDL: very low density lipoproteins.

## Authors' contributions

JP conducted lipoprotein isolation, culture of primary human macrophages and THP-1 cells, lipid and protein analyses as well as various ELISAs and real time PCR.

MWL performed cell viability assessment, free fatty acid analysis, fatty acid turnover and acyl-CoA synthetase activity assay, various ELISAs and statistical analysis.

All three authors contributed in the writing of the manuscript and participated in planning of the study design. All authors read and approved the final manuscript.

## References

[B1] Khovidhunkit W, Kim MS, Memon RA, Shigenaga JK, Moser AH, Feingold KR, Grunfeld C (2004). Effects of infection and inflammation on lipid and lipoprotein metabolism: mechanisms and consequences to the host. J Lipid Res.

[B2] Persson J, Degerman E, Nilsson J, Lindholm MW (2007). Perilipin and adipophilin expression in lipid loaded macrophages. Biochem Biophys Res Commun.

[B3] Guyton JR, Klemp KF (1994). Development of the atherosclerotic core region. Chemical and ultrastructural analysis of microdissected atherosclerotic lesions from human aorta. Arterioscler Thromb.

[B4] Ruan XZ, Moorhead JF, Fernando R, Wheeler DC, Powis SH, Varghese Z (2003). PPAR agonists protect mesangial cells from interleukin 1beta-induced intracellular lipid accumulation by activating the ABCA1 cholesterol efflux pathway. J Am Soc Nephrol.

[B5] Khovidhunkit W, Moser AH, Shigenaga JK, Grunfeld C, Feingold KR (2003). Endotoxin down-regulates ABCG5 and ABCG8 in mouse liver and ABCA1 and ABCG1 in J774 murine macrophages: differential role of LXR. J Lipid Res.

[B6] Grunfeld C, Feingold KR (1996). Regulation of lipid metabolism by cytokines during host defense. Nutrition.

[B7] Kruth HS, Skarlatos SI, Gaynor PM, Gamble W (1994). Production of cholesterol-enriched nascent high density lipoproteins by human monocyte-derived macrophages is a mechanism that contributes to macrophage cholesterol efflux. Journal Of Biological Chemistry.

[B8] Zhang WY, Gaynor PM, Kruth HS (1996). Apolipoprotein E produced by human monocyte-derived macrophages mediates cholesterol efflux that occurs in the absence of added cholesterol acceptors. Journal Of Biological Chemistry.

[B9] Duan H, Li Z, Mazzone T (1995). Tumor necrosis factor-alpha modulates monocyte/macrophage apoprotein E gene expression. J Clin Invest.

[B10] Zuckerman SH, Evans GF, O'Neal L (1992). Cytokine regulation of macrophage apo E secretion: opposing effects of GM-CSF and TGF-beta. Atherosclerosis.

[B11] Minor LK, Rothblat GH, Glick JM (1989). Triglyceride and cholesteryl ester hydrolysis in a cell culture model of smooth muscle foam cells. J Lipid Res.

[B12] Galea J, Armstrong J, Gadsdon P, Holden H, Francis SE, Holt CM (1996). Interleukin-1 beta in coronary arteries of patients with ischemic heart disease. Arterioscler Thromb Vasc Biol.

[B13] Barath P, Fishbein MC, Cao J, Berenson J, Helfant RH, Forrester JS (1990). Detection and localization of tumor necrosis factor in human atheroma. Am J Cardiol.

[B14] Niessner A, Shin MS, Pryshchep O, Goronzy JJ, Chaikof EL, Weyand CM (2007). Synergistic proinflammatory effects of the antiviral cytokine interferon-alpha and Toll-like receptor 4 ligands in the atherosclerotic plaque. Circulation.

[B15] Lindholm EM, Palmer AM, Graham A (2001). Triacylglycerol-rich lipoproteins alter the secretion, and the cholesterol-effluxing function, of apolipoprotein E-containing lipoprotein particles from human (THP-1) macrophages. Biochem J.

[B16] Rumsey SC, Galeano NF, Arad Y, Deckelbaum RJ (1992). Cryopreservation with sucrose maintains normal physical and biological properties of human plasma low density lipoproteins. J Lipid Res.

[B17] Maor I, Aviram M (1999). Macrophage released proteoglycans are involved in cell-mediated aggregation of LDL. Atherosclerosis.

[B18] Ohlsson BG, Englund MC, Karlsson AL, Knutsen E, Erixon C, Skribeck H, Liu Y, Bondjers G, Wiklund O (1996). Oxidized low density lipoprotein inhibits lipopolysaccharide-induced binding of nuclear factor-kappaB to DNA and the subsequent expression of tumor necrosis factor-alpha and interleukin-1beta in macrophages. J Clin Invest.

[B19] Persson J, Nilsson J, Lindholm MW (2006). Cytokine response to lipoprotein lipid loading in human monocyte-derived macrophages. Lipids Health Dis.

[B20] Sjoblom L, Eklund A (1990). Dietary protein and fatty acid composition of liver lipids in the rat. Biochim Biophys Acta.

[B21] Tanaka T, Hosaka K, Numa S (1981). Long-chain acyl-CoA synthetase from rat liver. Methods Enzymol.

[B22] Sambrook J, Fritsch EF, Maniatis T (1989). Molecular cloning, a laboratory manual.

